# A new paradigm for macromolecular crystallography beamlines derived from high-pressure methodology and results

**DOI:** 10.1107/S0909049510041695

**Published:** 2010-11-12

**Authors:** Roger Fourme, Eric Girard, Anne-Claire Dhaussy, Kadda Medjoubi, Thierry Prangé, Isabella Ascone, Mohamed Mezouar, Richard Kahn

**Affiliations:** a Synchrotron SOLEIL, BP 48, Saint Aubin, 91192 Gif-sur-Yvette, France; b IBS (UMR 5075 CEA-CNRS-UJF-PSB), 41 rue Jules Horowitz, 38027 Grenoble Cedex, France; c CRISMAT, ENSICAEN, 6 Boulevard du Maréchal Juin, 14000 Caen, France; d LCRB (UMR 8015 CNRS), Université Paris Descartes, Faculté de Pharmacie, 4 avenue de l’Observatoire, 75270 Paris, France; e ENSCP (UMR CNRS 7223), 11 rue Pierre et Marie Curie, 75231 Paris Cedex 05, France; f ESRF, BP 220, 38043 Grenoble, France

**Keywords:** macromolecular crystallography, high pressure, short X-ray wavelength

## Abstract

Biological structures can now be investigated at high resolution by high-pressure X-ray macromolecular crystallography (HPMX). The number of HPMX studies is growing, with applications to polynucleotides, monomeric and multimeric proteins, complex assemblies and even a virus capsid. Investigations of the effects of pressure perturbation have encompassed elastic compression of the native state, study of proteins from extremophiles and trapping of higher-energy conformers that are often of biological interest; measurements of the compressibility of crystals and macromolecules were also performed. HPMX results were an incentive to investigate short and ultra-short wavelengths for standard biocrystallography. On cryocooled lysozyme crystals it was found that the data collection efficiency using 33 keV photons is increased with respect to 18 keV photons. This conclusion was extended from 33 keV down to 6.5 keV by exploiting previously published data. To be fully exploited, the potential of higher-energy photons requires detectors with a good efficiency. Accordingly, a new paradigm for MX beamlines was suggested, using conventional short and ultra-short wavelengths, aiming at the collection of very high accuracy data on crystals under standard conditions or under high pressure. The main elements of such beamlines are outlined.

## Abstract 2

Macromolecular crystallography at high pressure (HPMX) is a mature technique. Shorter X-ray wavelengths increase data collection efficiency on cryocooled crystals. Extending applications and exploiting spin-off of HPMX will require dedicated synchrotron radiation beamlines based on a new paradigm.

## 1. Introduction

High-pressure (HP) molecular biophysics is a developing field. Our planet is generating a piezophilic environment since most of the biosphere is characterized by pressure greater than 10 MPa (100 MPa  = 0.1 GPa  = 1 kbar). The recent discovery of the first obligate piezophile archae, *Pyrococcus yayanosii* (Zeng *et al.*, 2009 ▶), illustrates the interest of high-pressure life adaptation. Concomitant to the understanding of life adaptation to high pressure, the question of the origin of life is raised. The potential throughput for biotechnological developments as well as for environmental questions is of societal interest. Pressure perturbation is a mild and efficient tool for exploring the whole spectrum of conformers of proteins and other biomolecules, from the native folded state to unfolded states. On the basis of differences in partial specific volume, higher-energy conformers of biological relevance can be selectively promoted by high pressure (Akasaka, 2006 ▶).

A better understanding of the fundamental mechanisms responsible for the effects of HP on biomolecules requires high-resolution structural information, which is still scarce. For a few years, such information has become accessible with the implementation of pressure perturbation in both NMR and macromolecular crystallography (MX). Up to 2002, only few crystal structures of small proteins at high pressure were published, using a beryllium cell (Kundrot & Richards, 1987 ▶; Urayama *et al.*, 2002 ▶). The lack of structural data at high pressure was due mainly to the cumulated complexities of high-pressure containment and crystallography and to the lack of interest in such studies. A technical breakthrough was achieved with a set-up at the ESRF ID30 then ID27 beamlines, combining a diamond-anvil cell (DAC), ultra-short-wavelength X-rays from undulators and a large area detector (Fourme *et al.*, 2001 ▶). The accessible pressure range was increased by almost one order of magnitude with respect to beryllium cells, exceeding 2 GPa. The quality of diffraction data collected under high pressure achieved usual standards (Girard *et al.*, 2007*a* ▶).

The main technical advances of high-pressure macromolecular crystallography (HPMX) will be recalled and a summary description of recent scientific results will illustrate its contribution to structural studies and for determining crystal and molecular compressibility, demonstrating that it can now be considered as a mature and general technique (Fourme *et al.*, 2009 ▶). HPMX instrumentation and methods have contributed to a renewed interest for short and ultra-short wavelengths,^1^ not only for HP studies but also for standard data collection. It is shown that, in addition to well documented advantages of these unusual wavelengths, the data collection efficiency (DCE), defined as the amount of diffraction data of a given resolution that can be acquired per crystal unit-volume (Fourme *et al.*, 2003 ▶), increases at shorter wavelengths. These results consolidate the scientific case for building synchrotron radiation beamlines dedicated to short-wavelength data collection and HPMX.

## 2. HPMX instrumentation

Two crucial components of a HPMX set-up are (i) the high-pressure cell and (ii) an intense, parallel (or slightly convergent) and highly monochromatic X-ray beam of short or, better, ultra-short wavelength. Recent high-pressure cells for HPMX are purposely designed DACs. They feature a large useful opening angle (∼85°) for both incoming and diffracted beams and the thrust applied to diamonds is generated by a pneumatic built-in system, either a membrane (Girard *et al.*, 2007*a* ▶) or a piston (Girard *et al.*, 2010*a* ▶) connected to an external source of pressurized gas. A single crystal with dimensions up to 350 × 350 × 150 µm, or alternatively several smaller crystals, can be compressed from atmospheric pressure to >2 GPa; temperature is adjustable from 293 to 393 K (Girard *et al.*, 2010*a* ▶). Currently, routine HPMX data collection with synchrotron radiation is available only at the 6 GeV ESRF storage ring, on the ID27 beamline and more recently FIP-BM30A. ID27 is a high-pressure diffraction beamline with a pair of in-vacuum undulators. As this multipurpose beamline is overbooked, only a small fraction of available beam time is allocated to HPMX, which limits the number of accepted proposals and practically excludes methodological programs requiring systematic tests. FIP-BM30A is a French CRG MX beamline on a bending magnet. Albeit not optimal for HPMX in terms of maximum useful photon energy, brightness and divergence, an interesting feature of this beamline is a six-axis robotic arm normally used as an automated sample changer. Mechanics and software were modified in order to handle the DAC, including automatic centring of the compressed crystal. The robotic arm acts as a goniometer for data collection by the oscillation method. Successful results were recently achieved (Girard *et al.*, 2010*a* ▶).

The third most important component of a HPMX set-up is the area detector. On FIP-BM30A, data were collected at short wavelength (photon energy ≃ 18 keV) using a Quantum 315R CCD detector (ADSC, USA). Experiments on ID27 at ultra-short wavelengths were initially performed using a Mar345 imaging plate detector (Marresearch, Germany). The large area of this detector allows the crystal-to-detector distance to be increased for a given resolution, which, with a parallel X-ray beam, improves the signal-to-noise ratio at a given wavelength (Schiltz *et al.*, 1997 ▶). Another useful characteristic of the imaging plate is a sensitive coating that contains barium. By adjusting the photon energy just over the Ba *K*-edge at 37.414 keV, the detector acts as an energy filter because the detective quantum efficiency (DQE) is maximal for elastic scattering and reduced for Compton scattering (Fourme *et al.*, 2001 ▶). Good results were obtained with this detector, albeit that the long readout time is a major drawback for HPMX data collection. The imaging plate was replaced by a Mar165 CCD detector (Rayonix, USA), which approximately doubled the data collection rate. The DQE of the Mar165 CCD detector with the standard phosphor^2^ is ∼0.55 and ∼0.14 at 18 and 33 keV, respectively (data from Rayonix). All photons shining on the sample contribute to sample degradation, but only a fraction (given by the detector DQE) is useful for diffraction. With a low-DQE detector, crystals are considerably over-irradiated and submitted during longer exposure times to damages from radicals produced by irradiation.

## 3. HPMX results

**Figure 1:**
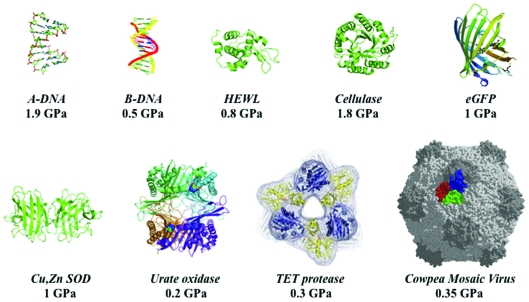
Selected examples of macromolecular structures investigated by HPMX (from works by authors of this article and co-workers).

As a general rule, any good quality macromolecular crystal loaded in the DAC can be compressed without degradation of three-dimensional order, up to a certain (crystal-specific) range of pressure where diffraction is reduced and finally lost. This remarkable behaviour was attributed to the peculiar nature, half-liquid and half-solid, of macromolecular crystals, which ensures substantial plasticity and hydrostatic compression of molecules thanks to solvent channels in the crystal. We have compressed crystals of various polypeptide, proteins and macromolecular assemblies, some of them beyond 1 GPa and sometimes up to 2 GPa, without substantial reduction of diffracting power (Fig. 1 ▶, Table 1 ▶). Observed effects of pressure include (i) elastic compression of the native sub-state (Girard *et al.*, 2005 ▶), (ii) higher-energy sub-states of biological interest where modifications of the native state were of small amplitude, mainly at the active site (Girard *et al.*, 2010*b* ▶; Ascone *et al.*, 2010*b* ▶), (iii) dissociation of an oligomeric protein (Girard *et al.*, 2010*b* ▶) and (iv) anisotropic compression of protein backbone and of side chains (Ascone *et al.*, 2010*b* ▶). HPMX has also been used to measure, in addition to crystal compressibility, the intrinsic molecular compressibility of several polypeptides and proteins (Ascone *et al.*, 2010*a* ▶).

The accuracy and completeness of diffraction data as well as refinement results have reached in most cases standards of conventional MX (Table 1 ▶).

## 4. Shorter wavelengths increase data collection efficiency

The interest for macromolecular crystallography in short and ultra-short wavelengths emitted by bright synchrotron sources was strongly advocated by Helliwell *et al.* (1993 ▶), who showed that data sets of unprecedented quality could be obtained at these wavelengths by reducing random and systematic errors in conjunction with very high values for completeness and multiplicity. Gonzalez *et al.* (1994 ▶) did not find improvement in the signal-to-noise ratio at 0.55 Å with respect to 0.92 Å. Schiltz *et al.* (1997 ▶) performed the first complete experiment at ultra-short wavelength, exploiting anomalous scattering near the Xe *K*-edge for phasing. Fourme *et al.* (2003 ▶), in the context of the use of ultra-short wavelengths in particular for HPMX, discussed ways to improve the signal-to-noise ratio in a particular diffraction experiment, in order to increase the DCE. Whether the DCE is improved at higher energy for data collected under essentially the same experimental conditions on a given sample remained a matter of controversy.

**Figure 2:**
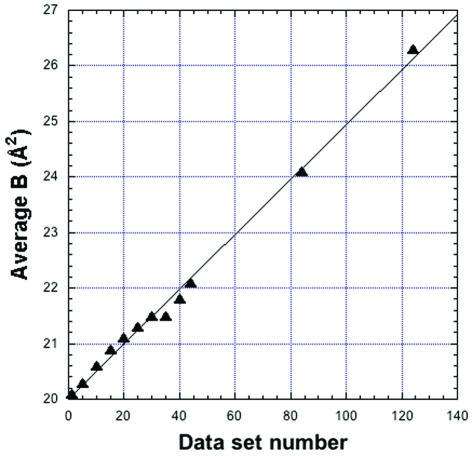
Variation of the average *B* factor as a function of data set number during multiple data acquisition with 18 keV photons and a CCD detector.

**Figure 3:**
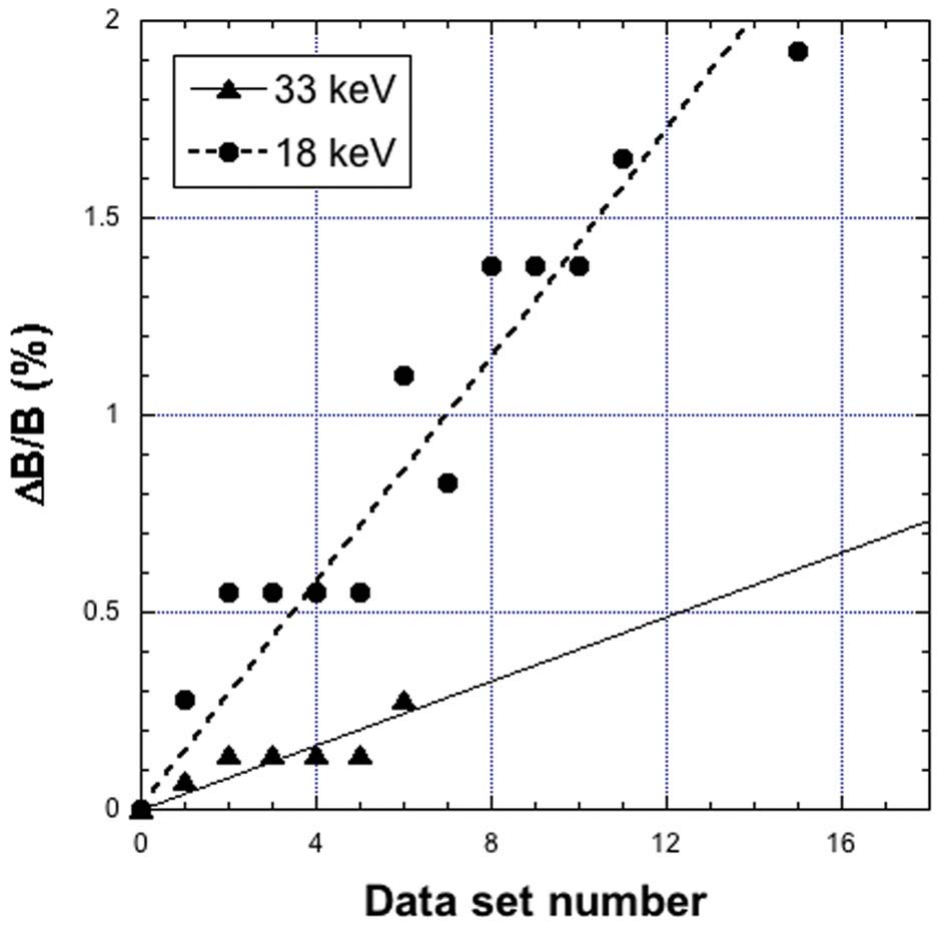
Intrinsic Δ*B*/*B* variation as a function of data set number during repeated acquisitions on a cryocooled HEWL crystal at 18 and 33 keV. The first data set is labelled 0 and the 18 keV plot is limited to the first 16 data sets of the complete collection shown in Fig. 2 ▶.

We have investigated this question from a practical point of view on cryocooled crystals at atmospheric pressure. Hen egg-white lysozyme (HEWL) crystals (space group *P*4_3_2_1_2) were grown at 293 K in a solution containing NaCl (1.6 *M*) and sodium acetate buffer (100 µL) pH 4.5. Samples from the same batch and with similar dimensions were mounted in loops and cryocooled in a stream of cold nitrogen at 100 K. Experiments were performed on the ID27 beamline using unfocused monochromatic beam from the Si(111) channel-cut monochromator adjusted at the zirconium or iodine *K*-edge (17.997 keV and 33.168 keV, respectively). The crystal-to-detector distance of the Mar165 CCD detector was set to obtain the same resolution at the detector edge for both energies. Data sets were collected over 90° in 1° steps. Exposure times were adjusted in order to collect data with the same average signal-to-noise ratio and resolution [1.5 Å at *I*/σ(*I*) ≥ 2]. At 18 keV, 44 sets were successively collected on the same crystal. While rotating over the same full angular range, the sample was irradiated for a time corresponding to the collection of ∼40 additional data sets, and a full data set was then recorded. The same procedure was repeated once. Data were integrated using the program *XDS* (Kabsch, 1993 ▶) and average *B*-factor values were obtained from Wilson plots calculated by the program. In Fig. 2 ▶, *B* values are plotted as a function of data set number for the complete data collection at 18 keV, showing a linear increase over a total irradiation corresponding to ∼124 data sets. At 33 keV, owing to longer exposure time and limited available beam time, seven data sets only were collected, showing also a linear increase of *B* values. Interestingly, slopes at 18 and 33 keV, 0.226 and 0.285% per data set, respectively, are similar in spite of the fact that the DQE at 33 keV is divided by ∼4 with respect to 18 keV (see §2). In Fig. 3 ▶, experimental Δ*B*/*B* values were multiplied by the detector DQE at the relevant wavelength, which gives the (intrinsic) variation which would be observed with an ideal detector (DQE = 1). The slopes of fitted linear curves in Fig. 3 ▶ are 0.041 and 0.143% per data set at 33 and 18 keV, respectively. Accordingly, assuming an ideal detector, the number of data sets of the prescribed quality that could be collected on a single HEWL crystal would be multiplied by ∼3.5 at 33 keV when compared with 18 keV. The actual intrinsic gain at 33 keV is probably even larger, given that longer exposures (necessary to compensate for the low DQE of the detector at 33 keV) increase the residence time in the sample of aggressive radicals produced by irradiation.

**Figure 4:**
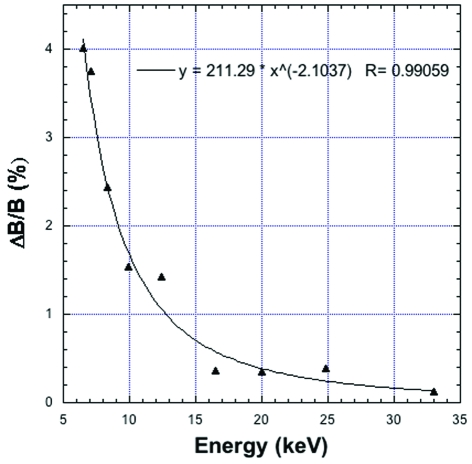
Variation of Δ*B*/*B* per data set *versus* energy during repeated data acquisitions on a cryocooled HEWL crystal performed at nine photon energies from 6.5 to 33 keV with a CCD detector. Analysis is based on Table 1 of Shimizu *et al.* (2007 ▶), after rescaling assuming DQE  =  1 over the whole energy range.

Shimizu *et al.* (2007 ▶) have reported measurements of successive 180° data sets (12 to 15) collected on a cryocooled HEWL crystal at nine X-ray energies from 6.5 to 33 keV using a Quantum 315 (ADSC, USA), a multi-CCD detector with the same type of phosphor as the Mar165 CCD. These authors were mainly interested in the dose dependence of radiation damage with photon energy. At each energy, average *B* factors were derived from a Wilson plot for the first and last data set. We analyzed these data assuming again DQE = 1. We found that the relative variation of *B* per data set was decreasing from 6.5 keV to 33 keV (Fig. 4 ▶), thus consolidating and extending to a much broader energy range conclusion drawn from our measurements at 18 and 33 keV. Systematic and random errors during data collection are smaller when using shorter wavelengths (Helliwell *et al.*, 1993 ▶; Fourme *et al.*, 2003 ▶). Results presented in this article for HEWL crystals suggest that, in addition, more data can be collected on a given cryocooled sample, which gives access to, for example, longer exposures, collection over a broader angular range and/or repeated acquisitions. These various effects of shorter wavelengths converge to improve data accuracy. Experiments using anomalous scattering from elements with absorption edges at ultra-long wavelengths (*e.g.* S or P) would benefit from the higher accuracy of conventional short or ultra-short wavelength data, whereas the lower accuracy of data collected at ultra-long wavelengths would essentially cancel the interest of stronger anomalous signals. The optimal wavelength for MX experiments is a matter of discussion. We suggest that the range 0.3–0.4 Å is the best choice (albeit shorter wavelengths may be useful for MAD phasing), considering both the increasing ratio of Compton scattering to elastic scattering with photon energy and the current detector technology.

Exploiting the full potential of ultra-short wavelengths would require detectors with a much better DQE than popular MX detectors optimized for conventional wavelengths, such as silicon pixel detectors, or standard CCD detectors with Gd_2_O_2_S:Tb phosphor. Increasing the thickness of this phosphor coating by a factor of two would increase the DQE by about the same factor, at the expense of a degradation of spatial resolution. The high-pressure beamline ID09A at ESRF (M. Hanfland, private communication) is operating with a Mar555 detector where photons are converted in a fairly thick selenium layer. Such detectors will require further evaluation for MX. Modular CdTe pixel detectors (Cd and Te *K*-edges at 26.727 and 31.817 keV, respectively) are promising. The measured DQE at 35 keV on a prototype 2 cm × 3 cm imager is ∼0.8 (Medjoubi *et al.*, 2010 ▶). Larger detectors are being developed.

Measurements of the degradation of irradiated cryocooled crystals probe essentially effects of primary damage. The behaviour of crystals may be very different at room temperature, and we plan to repeat our experiments on HEWL crystals at room temperature and perform similar measurements on other biomolecular crystals, both cryocooled and at room temperature.

## 5. Components of a beamline dedicated to both conventional and high-pressure MX data collection at short wavelength

MX beamlines can be found at all synchrotron radiation facilities, except those with a low-energy storage ring. The design goals of recent MX beamlines are: (i) wavelength tunability, in order to exploit anomalous phasing, in particular of selenium atoms, albeit that some beamlines are operating at an essentially fixed wavelength; (ii) large CCD or pixel detectors with a good DQE at conventional wavelengths; (iii) automated sample changer; and (iv) remotely controlled operation. So, most beamlines are, not surprisingly, quite similar. Beamlines designed to collect data from microcrystals have some tighter requirements, in particular for overall mechanical stability, goniometer accuracy, focusing optics and sample viewing.

The proposed beamline, based on a different paradigm, is designed for data collection using short- and ultra-short-wavelength X-rays on crystals in either standard conditions (*i.e.* at ambient pressure) or under high pressure, while preserving access to the whole range of conventional wavelengths. This beamline would extend the possibility of optimized SAD and MAD measurements, encompassing^3^ *K*-edges of all elements with *Z* from 25 to 58 (*i.e.* from Mn to Ce). Short and ultra-short wavelengths contribute efficiently to increase the accuracy of measurements, and the beamline itself should be designed in order to contribute to this quest of ultimate accuracy.

*Source.* With in-vacuum undulators on high-energy storage rings (ESRF, SPring-8, APS, PETRA III), the shortest useful wavelength is below 0.3 Å, whereas with intermediate-energy machines, such as DIAMOND and SOLEIL, the practical limit is currently ∼0.4 Å. Topping-up mode, when available, contributes to high stability. A pair of undulators might be required to cover the whole energy range.

*Optics.* The main (and perhaps unique) optical element would be a monochromator producing a very stable and parallel beam with a narrow bandpass over the whole energy range. A simple solution could be based on a pair of translatable Si(111) and Si(311) channel-cut crystals. Mirrors or other focusing devices would be optional. Optics would be mounted on a heavy table with a vertical translation motion in order to compensate the beam height variation during wavelength change, as implemented on, for example, BM14 at ESRF and PROXIMA 1 at SOLEIL.

*Experimental set-up.* For conventional MX, the most flexible solution, particularly for measuring accurately anomalous pairs and exploiting the anisotropy of anomalous scattering (Schiltz & Bricogne, 2008 ▶), is a kappa goniometer. In the high-pressure mode, the weight and bulk of the DAC are hardly compatible with the kappa goniometer and a second goniometer would be required with a single-axis rotation, preferably about a vertical axis, because in this geometry motions of the crystal in the compression cavity of the DAC are minimal during data collection. Using the six-axis robotic arm (normally used as sample changer) to handle the DAC and act as goniometer would be an elegant solution allowing to switch readily from normal operation (with the kappa goniometer) to high-pressure mode (Girard *et al.*, 2010*a* ▶).

Two items are required for high-pressure experiments: an optical system, coaxial with the X-ray beam, ensuring both the collection of ruby fluorescence signal and sample viewing (*e.g.* PRL from Betsa, Nangis, France); and a device for programmed pressure ramping or pressure cycling (in view of sample annealing), with monitoring based on the actual pressure in the compression cavity. This system is being tested on the ESRF ID27 beamline.

*Detector.* The main characteristics of a detector for this beamline would be a good DQE over the whole energy range, a low intrinsic noise, a large area (>30 cm × 30 cm) and fast readout.

## 6. Summary and conclusions

HPMX is now a method that is technically mature and has been applied to a variety of biomolecular crystals. In particular, high pressure can be used to promote and trap higher-energy conformers of biological relevance, and the feasibility of such studies in the crystalline state is being investigated.

The successful use of ultra-short wavelengths for HPMX led us to investigate the interest of wavelengths shorter than usual in conventional crystallography. Such wavelengths improve not only data quality but also the intrinsic (*i.e.* assuming an ideal detector) DCE. It is likely that these conclusions will extend to other cryocooled biomolecular crystals and higher photon energies. On the basis of present results, we suggest a new paradigm for synchrotron radiation beamlines. For such beamlines, the crucial importance of detectors with a reasonable efficiency at short wavelengths is underlined.

## Figures and Tables

**Table 1:** Results of HPMX studies where data collection at one or several pressures was completed *N* = number of crystals. Res = resolution. Compl = completeness. Red = redundancy. PDB = Protein Data Bank. IP = imaging plate.

System	Space group	Cell parameters *a*, *b*, *c* (Å)	λ (Å)/detector	*P* (GPa)	*N*	Res	*R*_merge_ (%)	*I*/σ	Compl (%)	Red	*R*_work_ (%)	*R*_free_ (%)	Reference	PDB ID
HEWL	*P*4_3_2_1_2	77.54, 77.54, 38.05	0.331/IP	0.30	1	1.98	4.8	19.1	79.5	4.2	18.8	22.0	Fourme *et al.* (2001 ▶)†	
	*P*4_3_2_1_2	76.77, 76.77, 37.80	0.331/IP	0.58	1	1.99	5.2	20.7	84.3	4.8	20.8	24.5	Fourme *et al.* (2001 ▶)†	
	*P*4_3_2_1_2	76.58, 76.58, 37.55	0.331/IP	0.69	2	1.81	7.6	21.4	70.6	3.9	22.8	26.1	Fourme *et al.* (2001 ▶)†	
UOX	*P*2_1_2_1_2_1_	79.70, 95.87, 104.99	0.374/CCD	0.15	1	1.8	5.2	7.4	96.4	2.7	17.8	21.8	Girard *et al.* (2010*b* ▶)	3f2m
SOD	*P*2_1_2_1_2_1_	46.85, 50.51, 146.38	0.331/IP	0.57	2	2.0	11.1	11.5	91.0	5.8	17.2	21.8	Ascone *et al.* (2010*b* ▶)	3hw7
CpMV	*I*23	313.38, 313.38, 313.38	0.331/IP	0.33	8	2.8	14.9	4.9	91.2	3.4	16.3	17.1	Girard *et al.* (2005 ▶)	
Cellulase	*P*2_1_2_1_2_1_	44.66, 78.87, 136.10	0.331/IP	1.75	2	1.80	–	–	–	–	–	–	Refinement in progress	
A-DNA	*P*6_1_	43.71, 43.71, 40.70	0.374/CCD	0.55	1	1.65	4.3	10.2	94.1	3.2	16.9	20.1	Girard *et al.* (2007*b* ▶)	2pl4
	*P*6_1_	43.17, 43.17, 40.38	0.374/CCD	1.04	1	1.60	5.8	6.6	89.4	3.2	18.9	22.1	Girard *et al.* (2007*b* ▶)	2pl8
	*P*6_1_	42.83, 42.83, 40.30	0.374/CCD	1.39	1	1.60	4.7	11.4	98.5	3.1	18.7	22.2	Girard *et al.* (2007*b* ▶)	2plb
B-DNA	*P*2_1_2_1_2_1_	25.23, 40.66, 65.20	0.374/CCD	0.35	2	2.55	11.5	8.1	78.5	3.2	20.5	27.1	Refinement in progress	
Z-DNA	*P*2_1_2_1_2_1_	17.77, 30.86, 43.45	0.8/CCD	0.72	3	1.55	–	–	–	–	–	–	Data collected	
eGFP	*C*222_1_	37.72, 130.37, 110.60	0.374/CCD	7.85	2	1.9	–	–	–	–	–	–	Data at four pressures, refinement in progress	

† Structure refinements by T. Prangé (private communication).

## Footnotes

1 Nomenclature: ultra-long wavelengths, λ ≥ 3 Å; long wavelengths, 2 Å ≤ λ < 3 Å; conventional wavelengths, 1 Å ≤ λ < 2 Å. Short wavelengths, 0.5 Å ≤ λ < 1 Å; ultra-short wavelengths, λ < 0.5 Å, as proposed by Helliwell *et al.* (1993 ▶).

2 Gd_2_O_2_S:Tb, thickness 40 µm, and assuming an effective density of 4.5 g cm^−3^.

3 Assuming a lower wavelength limit at 0.3 Å.
